# Glucagonoma and Glucagonoma Syndrome: An Updated Review

**DOI:** 10.1111/cen.15300

**Published:** 2025-07-04

**Authors:** Sofia Anelli, Rossella Mazzilli, Virginia Zamponi, Beatrice Giorgini, Bianca Golisano, Camilla Mancini, Flaminia Russo, Francesco Panzuto, Antongiulio Faggiano

**Affiliations:** ^1^ Endocrinology Unit, Department of Clinical and Molecular Medicine ENETS Excellence Center, Sapienza University of Rome, Sant'Andrea Hospital Rome Italy; ^2^ Department of Digestive Disease, ENETS Excellence Center Sant'Andrea University Hospital, Sapienza University of Rome Rome Italy

**Keywords:** glucagonoma, glucagonoma syndrome, necrolytic migratory erythema

## Abstract

**Background:**

Glucagonoma is a rare well‐differentiated slowly proliferating pancreatic neuroendocrine tumour, characterized by several manifestations including necrolytic migratory erythema, weight loss, diabetes and anaemia.

**Aim:**

The purpose of the current review was to acknowledge literature about this rare tumour discerning the clinical features, diagnosis, treatment and prognosis of glucagonoma by comparing three different periods.

**Results:**

A study published by Song reviewed 216 cases from studies published between 1999 and 2016, making a comparison with 407 cases reported before 1998 by Soga and Yakuwa. The current review consisted of 86 cases reported in studies published in and after 2017: 40 males and 46 females, with an average age of 48.2 years. The male‐to‐female ratio was 0.87. The rate of typical clinical findings was as follows: NME, 93.2% (69/74); DM, 70.3% (50/74); weight loss, 62.2% (46/74); anaemia, 44.6% (33/74); glossitis or stomatitis or cheilitis, 31.1% (23/74). A total of 85 cases reported the location of the tumour as the pancreas and 38.8% of these cases involved the tail of the pancreas. The average tumour size was 4.6 cm in patients between 2017 and 2024. Metastasis was detected in 52.3% of patients (45/86). The comparison with previous series highlighted an earlier age of diagnosis of glucagonoma and a higher rate of NME, consistent with a higher diagnostic accuracy.

**Conclusions:**

Glucagonoma is a rare pathology, with peculiar characteristics that need to be acknowledged to achieve a timely diagnosis and finally improve patient prognosis.

## 1 Introduction

Glucagonoma is a rare pancreatic neuroendocrine tumour (pNET) originating from the alpha cells of the pancreas, with a global incidence of approximately one in 20 million people per year [1]. Most glucagonomas occur sporadically, although a small percentage, around 3%, can be inherited and can be associated with other tumours in the context of multiple endocrine neoplasia (MEN) 1 [2]. Glucagonoma can cause a range of symptoms known as ‘glucagonoma syndrome’, including necrolytic migratory erythema (NME), diabetes mellitus (DM), weight loss, normochromic normocytic anaemia, diarrhoea/steatorrhea, venous thrombosis and neuropsychiatric disturbances [3].

In 1998, Soga and Yakuwa evaluated 407 reported cases of glucagonoma and in 2018 Song reported 216 cases of glucagonoma published between 1999 and 2016 [4, 5], highlighting the major characteristics of the syndrome.

The aim of the present review was to evaluate cases of glucagonoma documented in and after 2017 and make a comparison with the previous published series, to show the trend of the disease during the last decades.

## 2 Methods

### 2.1 Literature Search

A literature search was conducted using the following search key word ‘Glucagonoma’. Information was obtained through PubMed.

### 2.2 Inclusion and Exclusion Criteria

Studies published in and after 2017 were included in the current review if they met the following criteria: (a) patients with specific pathological diagnosis or even in absence of a pathological confirmation, the coexistence of glucagonoma syndrome and elevated serum glucagon level; (b) demographic and clinical features of the patients available. Studies were excluded if: (a) published in languages other than English; (b) were a duplicate publication; (c) no data available behind the title and abstract to be evaluated.

The comparison was done with cases published before 1998 (defined as series C) and between 1999 and 2016 (defined as series B). We will refer to cases after 2017 as series A.

## 3 Characteristics of Included Studies

A total of 147 articles published in and after 2017 were identified as relevant in PubMed. A total of 9 articles were excluded as they were not published in English. A total of 62 articles were excluded because they were reviews, duplicate publications, or not including or not accessible clinical information about glucagonoma. A total of 12 articles were excluded as not enough information about glucagonoma was provided. Therefore, 64 articles were included in the current review, documenting 86 cases of glucagonoma in and after 2017 that met the inclusion criteria (Flow chart 1).

**Figure 1:**
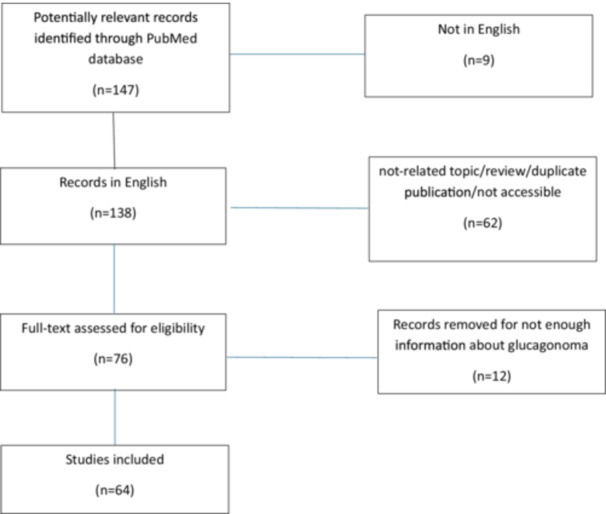
Flow chart of the included studies.

## 4 Results

### 4.1 Demographic Data

In series A, out of the 86 included cases 40 were males and 46 were females. The male‐to‐female ratio was 0.87 (as presented in Table 1). In series B, 89 were males and 111 were females and the ratio male‐to‐female was 0.80; in series C, males were 179 and females were 228 and the ratio was 0.79. Patient age was reported in 80 of the 86 patients and the average age was 48.2 years old. The average age of the patients documented in series B was 52.2 years old and the average age of patients in series C was 52.5 years (Table 2).

**Table 1:** Patient sex and male‐to‐female ratio.

	Glucagonoma	Male	Female	Male‐to‐female ratio
From 2017	86	40	46	0.87
Between 1999–2016	216 (200 with information on patient sex)	89	111	0.80
Before 1998	407	179	228	0.79

**Table 2:** Average age at diagnosis of glucagonoma and age range.

Average age and age range (years)		Range	Male	Female	Average
From 2017	86 (80 with data on patient sex)	14–77	50.7	45.7	48.2
Between 1999–2016	216 (200 with data on patient sex)	15–77	52.2	52.2	52.2
Before 1998	407	11–88	52.3	52.7	52.5

### 4.2 Clinical Manifestation

Regarding the clinical manifestations of glucagonoma, data were available in 74 patients of the 86 cases documented in series A, in 192 of series B and in 233 in series C.

The rate of frequency of NME was higher in series A compared to series B (93.2% vs. 79.2%; *p* = 0.0057). No significant difference in the other clinical manifestations were observed comparing series A with other groups (Table 3). In the current series, there was a small number of patients, 8, who were not diagnosed with DM but had prediabetes, defined as impaired fasting glucose (IFG), a fasting glucose of 100–125 mg/dL (5.6–6.9 mmol/L), and IGT, a 2‐h glucose level of 140–199 mg/dL (7.8–11.1 mmol/L) during an oral glucose tolerance test (OGTT) [6, 7, 8, 9, 10, 11, 12, 13]. If the DM and prediabetes were considered together, the rate was significantly higher in series A than in series B (81% vs. 62.5%; *p* = 0.005).

**Table 3:** Clinical manifestations of glucagonoma.

Clinical manifestations	Series A From 2017 (*n* = 74)	Series B Between 1999–2016 (*n* = 192)	Series C Before 1998 (*n* = 233)	*p*‐value
NME	69 (93.2%)	152 (79.2%)	198 (85.0%)	0.0057 vs. series B 0.0751 vs. series C
Diabetes	52 (70.3%)	120 (62.5%)	171 (73.4%)	NS
Weight loss	46 (62.2%)	104 (54.2%)	152 (65.2%)	NS
Anaemia	33 (44.6%)	84 (43.8%)	127 (54.5%)	NS
Cheilitis, glossitis, stomatitis	23 (31.1%)	83 (43.2%)	92 (39.5%)	NS

In series A we observed a difference between the clinical manifestations of glucagonoma according to sex: the rate of NME and anaemia was significantly higher in females (100% vs. 85.3% in males, *p*‐value 0.017 and 62.5% vs. 23.5% in males, *p*‐value 0.001, respectively). No significant differences in the other clinical manifestations were observed: the rate of DM was 73.5% in males and 67.5% in females. Onychodystrophy was only documented in males [14, 15]. Some patients had been reported to be suffering from mental symptoms, including depression [16, 17, 18, 19], lability of mood [20], bipolar affective disorder, insomnia [7, 21] and malaise [22]. Certain patients exhibited neurological symptoms, including headaches, left‐sided weakness/discoordination, motor and sensory deficits of the right extremities [11]. Some patients experienced onychia periungual [23], hypotrichosis [24] and alopecia, greyish hair discoloration [7, 14, 15]. Other rare symptoms reported included gingivitis and balanitis [25, 26].

In rare cases, glucagonoma may present as dilated cardiomyopathy: the treatment with octreotide showed minimal improvement in ejection fraction (27% from baseline of 20.9%) [11]. Rarely glucagonoma can show as cerebral vein thrombosis [27].

In series A, co‐secretion of hormones was detected in 18 cases out of 86: those producing insulin were detected in half of the cases (9, 50%); other hormones co‐produced were gastrin (44.4%), parathormone (22.2%), pancreatic polypeptide (11.1%), vasoactive intestinal polypeptide (5.6%), calcitonin (5.6%); finally, in 44.4% of cases, co‐secretion of multiple hormones was reported.

### 4.3 Tumour Site and Size

Table 4 presents information regarding tumour location. In series A, a total of 85 (98.8%) cases reported the tumour to be located within the pancreas; this prevalence was higher compared to series B and series C (72.7% and 84.0%, respectively; *p* < 0.0001).

**Table 4:** Primary tumour sites.

Primary lesion sites	Series A From 2017 (*n* = 86)	Series B Between 1999–2016 (*n* = 216)	Series C Before 1998 (*n* = 407)	*p*‐value
Pancreas	85 (98.8%)	157 (72.7%)	342 (84.0%)	< 0.0001
Head	14 (16.5%)	20 (12.7%)	63 (18.4%)	NS
Neck	1 (1.2%)	7 (4.5%)	0 (0.0%)	NS
Body	10 (11.8%)	18 (11.5%)	59 (17.3%)	NS
Tail	33 (38.8%)	57 (36.3%)	143 (41.8%)	NS
Head and neck	0 (0.0%)	3 (1.9%)	0 (0.0%)	NS
Head and body	3 (3.5%)	1 (0.6%)	7 (2.0%)	NS
Neck and body	0 (0.0%)	1(0.6%)	0 (0.0%)	NS
Body and tail	13 (15.3%)	47 (29.9%)	53 (15.5%)	NS
Head and tail	0 (0.0%)	3 (1.9%)	1 (0.3%)	NS
Diffuse	5 (5.9%)	0 (0.0%)	16 (4.7%)	0.0017 vs. series B 0.3884 vs. series C
Not specified	6 (7.1%)	55 (25.5%)	55 (13.5%)	0.0002 vs. series B 0.1061 vs. series C
Extrapancreatic organs	0 (0.0%)	3 (1.9%)	3 (0.7%)	NS
Not specified	1 (1.2%)	1 (0.5%)	7 (1.7%)	NS

Concerning pancreas, the vast majority in series A involved the tail (38.8%); the tail of the pancreas was the primary lesion site also in 36.3% of patients in series B and in 41.8% in series C; a diffused pattern of distribution of the lesions within pancreas was found in 5.9% of series A, 0% in series B (*p* = 0.0017) and 4.7% in series C; on the other hand, in series A, the exact site in the pancreas was unspecified in a lower percentage of cases (7.1% vs. 25.5% in series B; *p* = 0.0002).

As a whole, the vast majority had glucagonomas in the pancreas [28, 29, 30, 31, 32, 33, 34, 35, 36, 37, 38]; six had extra pancreatic lesions and in nine cases primary tumour site was unknown.

Tumour size was recorded in 83.7% (72/86) cases reported in series A [39, 40, 41, 42, 43, 44, 45, 46, 47, 48, 49, 50, 51, 52, 53, 54, 55, 56, 57, 58, 59, 60, 61, 62]. Average diameter was 4.6 cm, similarly to the average diameter of 5.0 cm reported by Song in series B [5, 63]; the median tumour size in patients evaluated in series C was 3.1–5.5 cm [4].

### 4.4 Metastatic Disease

Table 5 focused on the metastasis spread. In series A, metastatic disease was reported in 52.3% of patients; the rate of metastases in lymph nodes was higher compared to series B (46.7% vs. 21.4%; *p* = 0.0046) and in spleen compared to series C (4.4% vs. 0%; *p* = 0.0308). No significant differences in the other sites of involvement were observed. Overall, the most common site of involvement was liver (80.0% vs 82.1% in series B and 79.9% in series C). In series A, the rate of metastatic disease at presentation was higher compared to recurrent metastatic disease post initial primary surgery (40, 88.9% vs. 5, 11.1%, *p* = 0.001).

**Table 5:** Metastases: sites of involvement.

Patients with metastases	Series A From 2017 (*n* = 45, 52.3%)	Series B Between 1999–2016 (*n* = 84; 44.7%)	Series C Before 1998 (*n* = 209; 51.4%)	*p*‐value
Liver	36 (80.0%)	69 (82.1%)	167 (79.9%)	NS
Lymph nodes	21 (46.7%)	18 (21.4%)	79 (37.8%)	0.0046 vs. series B 0.3136 vs. series C
Bones	2 (4.4%)	6 (7.1%)	17 (8.1%)	NS
Mesentery/omentum/peritoneum	0 (0.0%)	0 (0.0%)	10 (4.8%)	NS
Lung	0 (0.0%)	0 (0.0%)	6 (2.9%)	NS
Adrenal	0 (0.0%)	1 (1.2%)	3 (1.4%)	NS
Gallbladder	1 (2.2%)	1 (1.2%)	0 (0.0%)	NS
Ileal	1 (2.2%)	1 (1.2%)	0 (0.0%)	NS
Spleen or the hilus of spleen	2 (4.4%)	6 (7.1%)	0 (0.0%)	0.7126 vs. series B 0.0308 vs. series C
Diaphragm	1 (2.2%)	0 (0.0%)	0 (0.0%)	NS
Multiple but not specified	0 (0.0%)	2 (2.4%)	0 (0.0%)	NS

The tumour grade was recorded in 55 of 86 patients: G1, 49.1% (27/55); G2, 43.6% (24/55); G3, 7.3% (4/55). Tumour grade was not significantly associated to the metastatic status of glucagonomas.

### 4.5 Treatment and Prognosis

In series A, treatments were described in 84 patients: the vast majority underwent surgery (83.3%) and 45.2% of patients were treated with somatostatin analogues (SSA). Other treatments described are chemotherapy in 10.7% of cases, everolimus in 7.1%, sunitinib in 4.8%. As it concerns PRRT, only three patients (3.6%) received that treatment; however, in another patient, it was suggested to initiate PRRT. Patients also received TAE/TACE (7.1%) and radiofrequency ablation (4.8%) for metastases.

Clinical outcome was measured as progression‐free survival (PFS) for patients with locally advanced or metastatic disease undergone to systemic therapy and recurrence‐free survival (RFS) for patients undergone radical resection. PFS was defined as the time from the 1st line treatment initiation to disease progression (assessed according to clinical practice at the time of diagnosis), last visit, death from any cause, or loss to follow‐up. RFS was defined as the time from radical surgery to disease recurrence (assessed according to clinical practice at the time of diagnosis), last visit, death from any cause, or loss to follow‐up. Overall survival (OS) was defined as the time from diagnosis to death from any cause.

OS was recorded in 14 patients with a mean OS of 52.4 months; RFS was recorded in 36 patients with a mean RFS of 43.6 months; PFS was recorded in 11 patients with a mean PFS of 24 months.

No statistically significant differences were found according to treatment experienced, probably due to the small number of patients for some subgroups.

## 5 Discussion

Glucagonoma is a rare NET, secreting glucagon, which originates from the alpha cells of the pancreas.

A functioning NET syndrome is defined by the presence of a clinical syndrome combined with biochemical evidence of inappropriately elevated hormonal level; some NET may secrete biologically inactive hormonal variants or bioactive hormones at insufficient levels to elicit symptoms, and these should consequently be classified as nonfunctioning [64]. Most glucagonomas are sporadic, rarely they can be inherited: around 3% of inherited glucagonomas can be associated with other tumours in the context of MEN1 [2]. In the current review, MEN1 was recorded in 13 cases (15.1%) in and after 2017 [65, 66, 67, 68, 69, 70, 71]. This finding is only reported in series C by Soga et al. consistent with a similar rate of MEN1 glucagonoma (13%). Furthermore, we observed an early average age at diagnosis in series A than previous ones underlining an improvement of disease knowledge and diagnostic procedures.

The major diagnostic criteria of glucagonoma syndrome are: (1) Imaging study confirming presence of pancreatic tumour; (2) elevated glucagon levels (> 1000 pg/dL); (3) NME; (4) personal history of MEN1 [72]. Most of these are nonspecific, except for the NME, which is pathognomonic for glucagonoma; but skin lesions are often mistakenly treated as dermatitis before arriving at the correct diagnosis of NME. This explains the delay in diagnosis with a median of 39 months after the development of NME [7].

NME is a distinctive, intensely erythematous skin rash that may appear before other systemic symptoms. Individual lesions are pruritic and painful, initially appearing as erythematous vesicles and bullae that evolve into patches or plaques with irregular borders, crusting, ulcerations and scaling. Hyperglucagonemia is central in the pathogenesis of NME, inducing a catabolic state that depletes zinc, amino acids, and essential fatty acids; consequently, NME can be classified as a deficiency dermatosis and it can improve up to 50% with somatostatin analogues, amino acid infusion, essential fatty acids, topic or oral zinc therapies, vitamins, minerals and antibiotics [2, 73]. Interestingly, the rate of NME was significantly higher in the last decade, probably due to the growing knowledge about this rare feature and thus due to the early recognition of the disease related to it.

In patients with glucagonoma, to have good control of hyperglycaemia, metformin together with a low‐glucose diet should be considered; recently, metformin, has also emerged to be potentially active as an agent in cancer chemoprevention and treatment [74].

Among the techniques used to identify the tumour, there are contrast‐enhanced CT scan, magnetic resonance imaging (MRI), Gallium‐DOTA positron emission tomography (PET); the diagnosis can be confirmed by needle biopsy of the primary tumour or metastatic lesions. A multicentre study retrospectively analysed the endosonographic characteristics of small pNETs (including glucagonoma): 94% of them appeared hypoechoic on B‐mode ultrasound. After intravenous injection of contrast agents, 90% of the pNETs showed hyperenhancement compared to the enhancement of the surrounding pancreatic parenchyma [72].

As it concerns SSTR positivity, it was reported in 30 out of 86 patients; they had SSTR positivity using Ga‐DOTATATE PET/TC, Octreoscan or immunohistochemistry. Although in Song's review there is no mention of receptor positivity, Soga reported that somatostatin receptor scintigraphy was recorded in only four patients and was effective for localization of lesions in all four, suggesting that these tests were not widely used before 1998 and that they have been much more commonplace in the last decades.

The most effective treatment for the tumour and NME is surgical removal; surgery is recommended even if complete removal may not be possible, as it can improve survival and enhance quality of life [75]; it has shown that, after resection of functional NET, patients without symptom improvement had a lower 3‐year RFS than patients who did experience symptom improvement (49.9% vs. 80.3%, *p* = 0.005) [76]. In patients with contraindications to surgery, chemotherapy, everolimus or sunitinib are considered [77]. Recently, in patients with advanced and progressive NET, peptide receptor radionuclide therapy (PRRT) has been successful [78]; Klosinska reported a case of metastatic glucagonoma treated with [177Lu] Lu‐DOTA‐TATE used as a second‐line treatment in progressive disease [79]. Zandee demonstrated that treatment with 177Lu‐DOTA‐TATE is an effective therapy resulting in radiological, symptomatic and biochemical response in a high percentage of patients with metastatic functioning pNETs [80]. In the present series, only three patients received PRRT and they all had metastatic glucagonoma. Of them, one patient had neoadjuvant PRRT for tumour‐shrinkage before surgery, the second one had PRRT in second line after surgery and the third one had a partial response with a near‐complete regression of skin lesions. However, in another patient with metastatic glucagonoma, it was suggested to initiate PRRT in case of poor response to SSA therapy.

As reported by 2023 ENETS guidelines [81] paper, the management of functioning pancreatic neuroendocrine tumours (Pan‐NETs) in patients with advanced, unresectable, or metastatic disease requires a comprehensive, multimodal approach. This includes supportive care, medical therapies, interventional procedures, and surgical options. Some of these strategies can also be applied temporarily in patients who are awaiting potentially curative surgery. The treatment of symptoms in patients with glucagonoma primarily relies on SSA. These drugs are generally effective in most advanced cases, regardless of their ability to shrink the tumour. In addition to SSA, amino acid infusions and zinc supplementation have been suggested as helpful therapies for the skin condition NME. Symptom relief has also been observed with PRRT: in one study, 71% of patients experienced early symptom control without any hormonal crises [80].

In the past, it was thought that patients with functioning Pan‐NETs had a better prognosis after surgery than those with nonfunctioning tumours, likely because functional tumours are diagnosed earlier due to their hormonal symptoms. However, a more recent and extensive analysis of over 2500 resected patients from the SEER database contradicts this idea, showing no significant difference in overall or cancer‐specific survival between functioning and nonfunctioning tumours [82].

To control liver metastases, surgery is the first choice if at least 30% of the liver will remain and if there is no evidence of unresectable extrahepatic metastases or, in unresectable cases, hepatic artery embolization, radiofrequency ablation and cryoablation have been reported [83]: Myrehaug et al. reported a case of metastatic glucagonoma treated with radiation therapy which decreased the size of the liver metastasis and the serum level of glucagon level [84]; in a patient reported by Yu, CT‐guided microwave ablation was performed to treat multiple low‐density hepatic lesions [85]. In selected patients with unresectable metastatic NET liver transplantation should be considered; Coppa et al. proposed a selection based on the Milan criteria [86].

The OS observed in our study was 52.4 months, which is significantly higher than the 32.1 months reported by Song. This improvement could be attributed to advances in diagnostic and therapeutic strategies adopted in recent years, as well as an increased focus on overall patient management, which has contributed to improved quality of life in subjects with glucagonoma. Today, functional techniques such as PET with 68Ga‐DOTA‐SSA are available, which significantly improve tumour localisation compared to traditional radiological approaches; the early use of SSA and locoregional therapies (e.g., surgery, PRRT, liver embolization) has replaced exclusively symptomatic approaches of the past, leading to improved quality of life and increased survival.

In conclusion, glucagonoma is a rare pathology, and it is crucial for clinicians to know the major features of this condition to shorten the time to diagnosis and increase the rate of successful treatment. Early detection can significantly improve patient prognosis, with both survival rates and quality of life varying widely depending on the stage at diagnosis [87].

## Author Contributions

S.A., R.M., and A.F. conceived the study. S.A. and R.M. wrote the manuscript. V.Z., B.Gi., B.Go., and C.M. contributed to the acquisition of data. F.P. and A.F. revised the manuscript. All Authors give final approval of the version to be published.

## Conflicts of Interest

The authors declare no conflicts of interest.
